# Induction of HSPA4 and HSPA14 by NBS1 overexpression contributes to NBS1-induced *in vitro *metastatic and transformation activity

**DOI:** 10.1186/1423-0127-18-1

**Published:** 2011-01-06

**Authors:** Chung-Yin Wu, Chih-Ta Lin, Min-Zu Wu, Kou-Juey Wu

**Affiliations:** 1 Department of Occupational Medicine, Far Eastern Memorial Hospital, Taipei County, 220, Taiwan; 2 Institutes of Biochemistry and Molecular Biology, National Yang-Ming University, Taipei 112, Taiwan; 3 Institute of Bioinformatics and Systems Biology, National Chiao Tung University, Hsin-Chu 30068, Taiwan

## Abstract

**Background:**

Nijmegen breakage syndrome (NBS) is a chromosomal-instability syndrome associated with cancer predisposition, radiosensitivity, microcephaly, and growth retardation. The NBS gene product, NBS1 (p95) or nibrin, is a part of the MRN complex, a central player associated with double-strand break (DSB) repair. We previously demonstrated that NBS1 overexpression contributes to transformation through the activation of PI 3-kinase/Akt. NBS1 overexpression also induces epithelial-mesenchymal transition through the Snail/MMP2 pathway.

**Methods:**

RT-PCR, Western blot analysis, *in vitro *migration/invasion, soft agar colony formation, and gelatin zymography assays were performed.

**Results:**

Here we show that heat shock protein family members, A4 and A14, were induced by NBS1 overexpression. siRNA mediated knockdown of HSPA4 or HSPA14 decreased the *in vitro *migration, invasion, and transformation activity in H1299 cells overexpressing NBS1. However, HSPA4 or HSPA14 induced activity was not mediated through MMP2. NBS1 overexpression induced the expression of heat shock transcription factor 4b (HSF4b), which correlated with the expression of HSPA4 and HSPA14.

**Conclusion:**

These results identify a novel pathway (NBS1-HSF4b-HSPA4/HSPA14 axis) to induce migration, invasion, and transformation, suggesting the activation of multiple signaling events induced by NBS1 overexpression.

## Introduction

Nijmegen breakage syndrome (NBS) is a chromosomal-instability syndrome associated with cancer predisposition, radiosensitivity, microcephaly, and growth retardation [1-3]. The NBS gene product, NBS1 (p95 or nibrin), is a part of the MRN complex, a central player associated with DNA double-strand break (DSB) repair [1,2]. NBS1 carries out its checkpoint functions when it is phosphorylated by ATM (ataxia-telangiectasia mutated) protein after ionizing radiation [4-6]. We previously demonstrated that c-MYC, a dominant oncoprotein, directly activates NBS1 expression [7]. The proliferation-inducing function of NBS1 is supported by the phenotypes of diminished expansion of the inner cell mass of mutant blastocysts (Nbs1 null) and cellular proliferation defects in Nbs1^**m/m **^mouse embryonic fibroblasts (MEFs) [8-10]. NBS1 overexpression induces/enhances transformation activity through the activation of PI 3-kinase/Akt [11], indicating that overexpression of NBS1 is an oncogenic event. Increased NBS1 expression is also a prognostic factor for advanced stage head and neck squamous cell carcinoma (HNSCC) [12]. NBS1 interacts with the p110 subunits of PI 3-kinase to activate PI 3-kinase activity [13]. All these results implicate that NBS1 overexpression may play a significant role in tumor progression and metastasis.

Epithelial-mesenchymal transition (EMT) is a process initially observed in embryonic development in which cells lose epithelial characteristics and gain mesenchymal properties such as increased migration and invasion [14,15]. EMT is a critical event for tumor progression and metastasis of late-stage cancers [14,15]. Among the different EMT regulators, Snail induces matrix metalloproteinase-2 (MMP2) expression and contributes to increased invasiveness through the inhibition of cell-cell adhesion [16]. We recently demonstrated that NBS1 overexpression induces EMT through the Snail/MMP2 pathway [17], supporting the role of NBS1 overexpression in tumor progression and metastasis.

HSPA4 (Apg-2, HSP70) is considered to be a member of the Hsp110 family since it has a chaperone-like activity similar to Hsp110 [18,19]. HSPA4 responds to acidic pH stress, is involved in the radioadaptive response, and is overexpressed in hepatocellular carcinoma [19-21]. HSPA14 (Hsp70L1, HSP70-4) is expressed in the lens of zebrafish, forms the mammalian ribosome-associated complex with MPP11, and acts as a Th1 adjuvant through activation of dendritic cells [22-25]. Heat shock transcription factor 4b (HSF4b) is derived through alternative splicing and acts as a transcription activator [26]. HSF4b could be activated by the MAPK/ERK pathway and also recruits Brg1 during the G1 phase to regulate downstream heat shock proteins [27,28].

In this report, we demonstrate that NBS1 overexpression induced the expression of two heat shock proteins, HSPA4 and HSPA14. siRNA mediated repression of HSPA4 or HSPA14 decreased the *in vitro *migration, invasion, and transformation activity of NBS1 overexpressing cells. Induction of HSPA4 and HSPA14 did not overlap with the MMP2 pathway previously shown [17]. HSF4b expression correlated with the expression of HSPA4 and HSPA14. These results demonstrate the distinct pathway induced by NBS1 overexpression to enhance the *in vitro *metastatic and transformation activity in contrast to the Snail/MMP2 pathway [17].

## Materials and methods

### Cell lines, plasmids, and transfections

The non-small cell lung cancer cell line H1299 and human head and neck squamous cell carcinoma cell line FADU was previously described [12,17]. The pHeBOCMVNBS plasmid were described [11,12]. The FADUNBS1 cell line was described [12]. The H1299NBS1 stable clones were generated by transfecting the pHeBOCMVNBS construct into H1299 cells. The pHeBOCMV or pSUPER plasmid was stably transfected into FADU cells to generate the vector control cell lines. The pSUPER-HSPA4i and pSUPER-HSPA14i plasmids were made as described [11,12]. The oligonucleotides inserted into the pSUPER plasmid were described in additional file 1.

### Western blot analysis, RNA purification and RT-PCR analysis

Western blot analysis was performed as described [11-13]. For Western blot analysis, 50 μg protein extracts from each clone were loaded to 10% SDS-PAGE gels and transferred to nitrocellulose filters. The filters were probed with an anti-NBS1 antibody [11-13] and an anti-ß-actin antibody was selected as a loading control. Signals were developed using an ECL chemiluminescence kit (Amersham Biosciences, U.K.). Trizol (Invitrogen Life Technologies, Carlsbad, CA) was used for RNA purification from cultured cells. One μg of RNA was used for cDNA synthesis followed by PCR to evaluate the mRNA expression of NBS1, HSPA4, HSPA14, MMP2, HSF4b, HSF1, and HSF2 in different cell lines. The primer sequences used in RT-PCR were described in additional file 1. Since the antibodies against HSPA4, HSPA14, and HSF4b are not commercially available, only RT-PCR analysis could be used to analyze the expression levels of these molecules.

### *In vitro *cell migration and invasion assay

The procedures were performed as described [29,30]. Briefly, eight- μm pore size Boyden chamber was used for *in vitro *migration and invasion assays. Cells (1 × 10^5^) in 0.5% serum-containing RPMI were plated in the upper chamber and 15% fetal bovine serum was added to RPMI 1640 in the lower chamber as a chemoattractant. For invasion assay, the upper side of the filter was covered with Matrigel (Collaborative Research Inc., Boston, MA)(1:3 dilution with RPMI). After 12 hours for migration assay or 24 hours for invasion assay, cells on the upper side of the filter were removed, and cells that remained adherent to the underside of membrane were fixed in 4% formaldehyde and stained with Hoechst 33342 dye. The number of migrated cells was counted using a fluorescence microscope. Ten contiguous fields of each sample were examined using a 40× objective to obtain a representative number of cells which migrated/invaded across the membrane.

### Soft agar colony formation assay

The stable clones were plated at three different cell density (5 × 10^3^, 10^4^, 2 × 10^4 ^or 2.5 × 10^3^, 5 × 10^3^, 10^4^) using standard assay conditions as mentioned except that 15% FCS was used [11,12]. Data shown are representative of two or more experiments from independent cell cultures.

### Gelatin Zymography

Gelatin zymography was carried by subjecting 5 μg of each conditioned media sample to 10% SDS-PAGE containing 0.1% gelatin (G-9382, Sigma-Aldrich Corp., St. Louis, MO) as previously described [17]. Gels were stained with Coomassie Brilliant Blue R-250 and then destained. Transparent bands identified at 72 kD (latent form of MMP2) and 66 kD (active form of MMP2) on the Coomassie blue background of the gel were considered positive for the presence of enzymatic activity.

### Statistical analysis

Pearson Chi-square or Fisher's exact tests were used for comparison of dichotomous variables between groups, and the independent Student's *t*-test was used to compare the continuous variables between two groups [17,29,30]. The level of statistical significance was set at 0.05 for all tests.

## Results

### NBS1 overexpression in H1299 cells increased *in vitro *migration and invasion activity and induced the expression of HSPA4 and HSPA14

**Figure 1:**
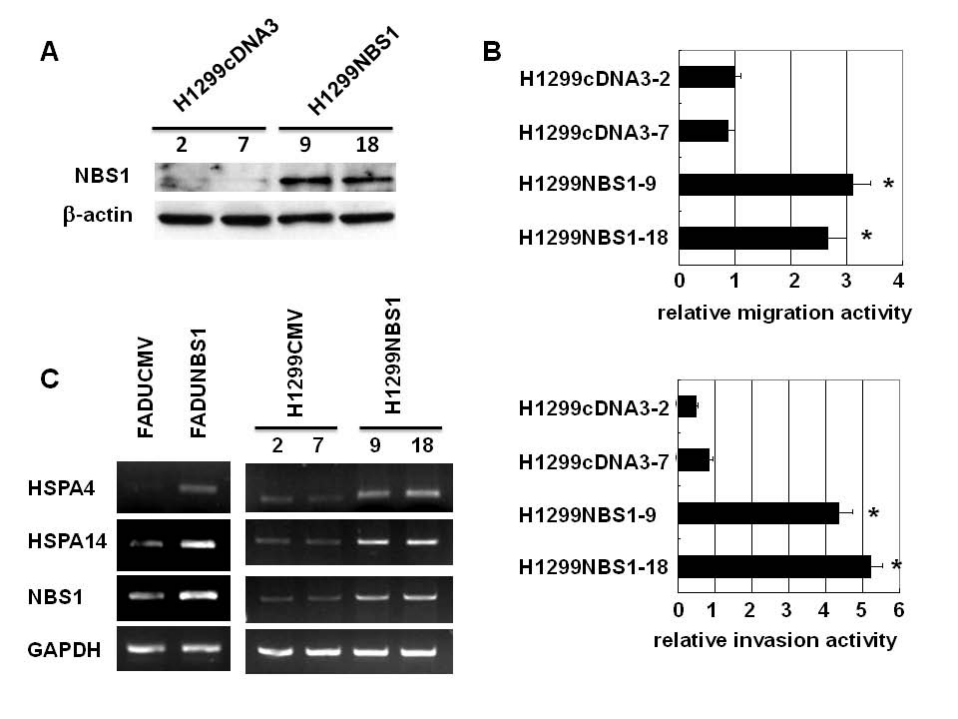
**NBS1 overexpression induced *in vitro *migration and invasion activity of H1299 cells and the expression of HSPA4 and HSPA14**. **(A) **Western blot analysis of NBS1 levels in H1299NBS1 clones vs. H1299 control clones. **(B) **NBS1 overexpression increased the migration and invasion activity of H1299 cells. The asterisk (*) indicated statistical significance (*P *< 0.05) between H1299NBS1 and H1299-control clones. **(C) **Induction of HSPA4 and HSPA14 expression by NBS1 overexpression in two different cell lines (FADU control vs. FADUNBS1, H1299 control vs. H1299NBS1).

We previously demonstrated that NBS1 overexpression induces epithelial-mesenchymal transition in head and neck cancer cell lines [17]. We wanted to test whether NBS1 overexpression could also increase *in vitro *migration and invasion activity in a lung cancer cell line. NBS1 expression vector was transfected into H1299 cells and stable clones were generated (Figure 1A). *In vitro *migration and invasion activity were measured in H1299NBS1 cell clones vs. the H1299 control clones. The results showed that NBS1 overexpression increased the *in vitro *migration and invasion activity compared with the control clones (Figure 1B).

To investigate the downstream signaling pathways responsible for NBS1-induced migration and invasion activity, a microarray approach was used to screen for genes activated by NBS1 overexpression. Among the numerous candidates, we focused on two heat shock proteins (HSPA4 and HSPA14) since many heat shock proteins such as HSP60, HSP90, GRP78, HSP27, and HSP70 were involved in metastasis [30-33]. In addition, these two proteins were not investigated to play a role in migration and invasion activity and may represent targets which have novel functions related to metastasis. To verify the results from microarray screening, a RT-PCR assay was performed to examine the increase in mRNA levels of HSPA4 and HSPA14. The result showed that the expression levels of HSPA4 and HSPA14 increased in two different NBS1 overexpressing cell lines (FADUNBS1 vs. FADU control; H1299NBS1 vs. H1299 control) (Figure 1C). These results confirmed the ability of NBS1 to induce *in vitro *metastatic activity in a lung cancer cell line and also identified the possible downstream targets of NBS1 overexpression.

### Knockdown of HSPA4 or HSPA14 decreased *in vitro *migration and invasion activity

**Figure 2:**
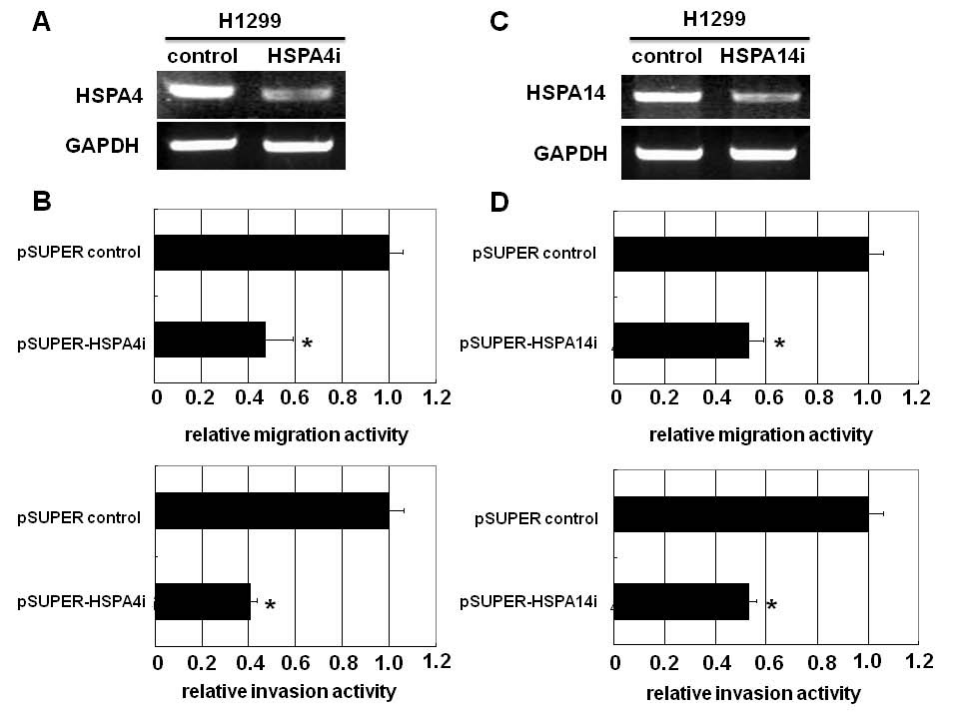
**Transient expression of siRNA against HSPA4 or HSPA14 in H1299 cells decreased the *in vitro *migration and invasion activity**. **(A) &(C) **Transient transfections of pSUPER-HSPA4i or pSUPER-HSPA14i decreased the expression of endogenous HSPA4 or HSPA14 in H1299 cells. **(B) & (D) **A significant decrease in migration and invasion activity in H1299 cells was shown by transient transfections of pSUPER-HSPA4i or pSUPER-HSPA14i vector into H1299 cells. The asterisk (*) indicated statistical significance (*P *< 0.05) between pSUPER vector control transfections vs. pSUPER-HSPA4i or pSUPER-HSPA14i transfections.

**Figure 3:**
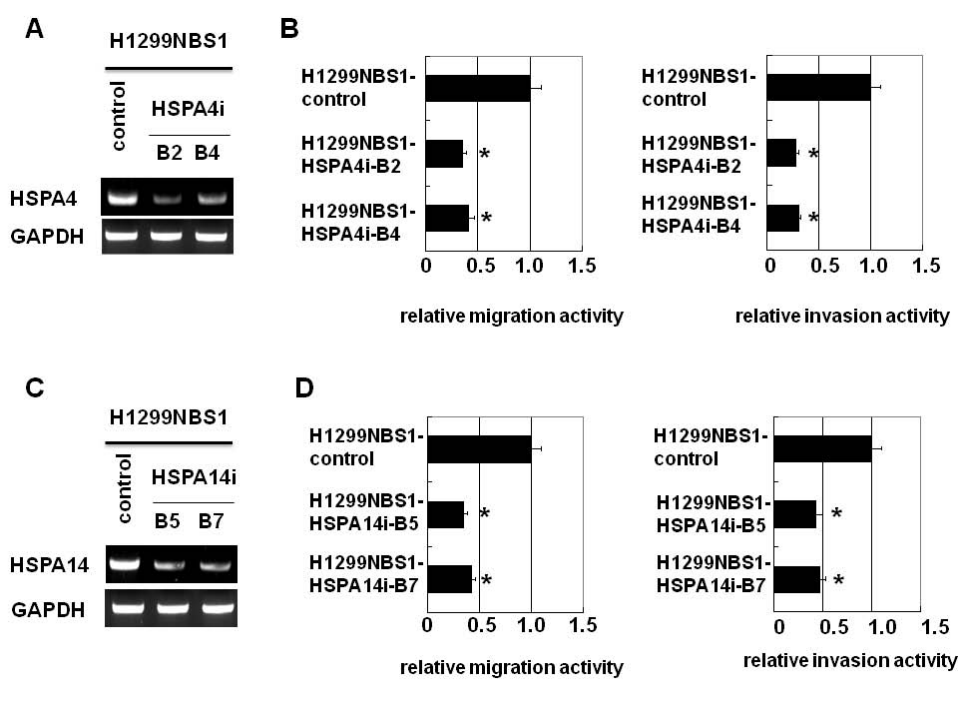
**H1299NBS1 stable clones expressing siRNA against HSPA4 or HSPA14 decreased the *in vitro *migration and invasion activity**. **(A) & (C) **H1299NBS1 clones stably transfected with pSUPER-HSPA4i or pSUPER-HSPA14i vector showed the decreased expression of endogenous HSPA4 or HSPA14 in H1299NBS1 cells. **(B) & (D) **A significant decrease in migration and invasion activity was shown in H1299NBS1 cells receiving siRNA against HSPA4 or HSPA14. The asterisk (*) indicated statistical significance (*p *< 0.05) between H1299NBS1 clones expressing siRNA and H1299NBS1 control clones.

To test the role of HSPA4 and HSPA14 in *in vitro *migration and invasion activity, siRNA-mediated repression of HSPA4 or HSPA14 was carried out in H1299 cells using transient transfection methods. The results showed that transient expression of siRNA against HSPA4 or HSPA14 caused a significant decrease in the mRNA levels of HSPA4 or HSPA14 in H1299 cells compared to the control-transfected cells (Figure 2A, C). *In vitro *migration and invasion activity also decreased in H1299 cells receiving siRNA to repress HSPA4 or HSPA14 (Figure 2B, D). To test the contribution of HSPA4 or HSPA14 to NBS1-induced migration and invasion activity, siRNA-mediated knockdown of HSPA4 or HSPA14 in H1299NBS1 cells was performed. Stable clones expressing siRNA against HSPA4 or HSPA14 were generated followed by the assay of *in vitro *migration and invasion activity. The results showed that knocking down HSPA4 or HSPA14 in H1299NBS1 cells caused a significant decrease in the *in vitro *migration and invasion activity, demonstrating the contribution of HSPA4 or HSPA14 to NBS1-induced migration and invasion activity (Figure 3B, D). However, knockdown of HSPA4 and HSPA14 in H1299 cells simultaneously did not further decrease the *in vitro *migration and invasion activity (additional file 2), suggesting the overlapping role of HSPA4 and HSPA14.

### Knockdown of HSPA4 or HSPA14 decreased *in vitro *transformation activity

**Figure 4:**
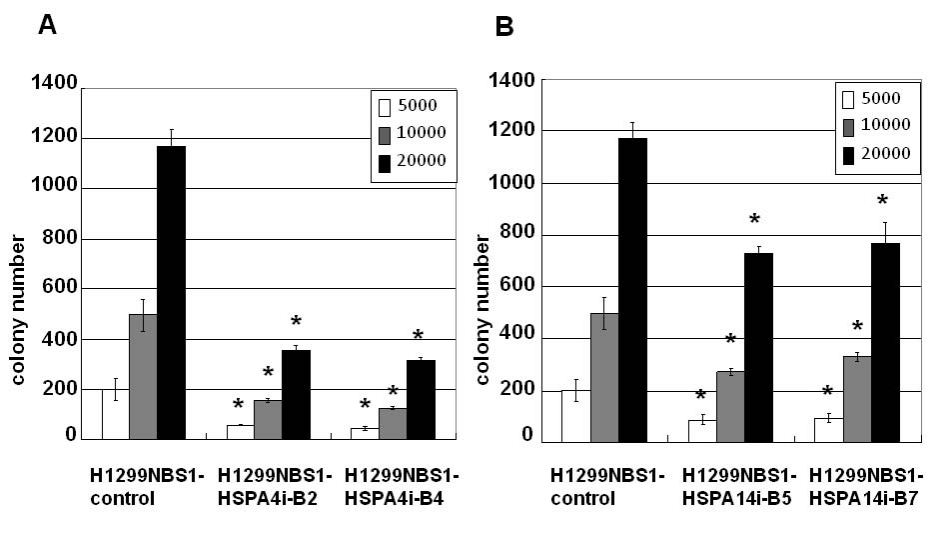
**H1299NBS1 clones expressing siRNA against HSPA4 or HSPA14 decreased the soft agar colony formation activity**. **(A) **&**(B) **A significant decrease in soft agar colony formation activity was shown in H1299NBS1 cells receiving siRNA against HSPA4 or HSPA14. The asterisk (*) indicated statistical significance (*p *< 0.05) between H1299NBS1 clones expressing siRNA and H1299NBS1 control clones.

We previously demonstrated that NBS1 overexpression contributes to transformation through the activation of PI 3-kinase/Akt [11]. To test the role of HSPA4 or HSPA14 in the transformation activity induced by NBS1, siRNA mediated repression of HSPA4 or HSPA14 in H1299NBS1 cells was performed (Figure 3A, C). The results showed a significant decrease in soft agar colony formation activity in H1299NBS1 stable clones expressing siRNA against HSPA4 or HSPA14 (Figure 4A, B), suggesting the contribution of HSPA4 or HSPA14 to *in vitro *transformation activity.

### Knockdown of HSPA4 or HSPA14 did not influence the expression or activity of MMP2 and the correlation of HSF4b expression with HSPA4/HSPA14 expression

**Figure 5:**
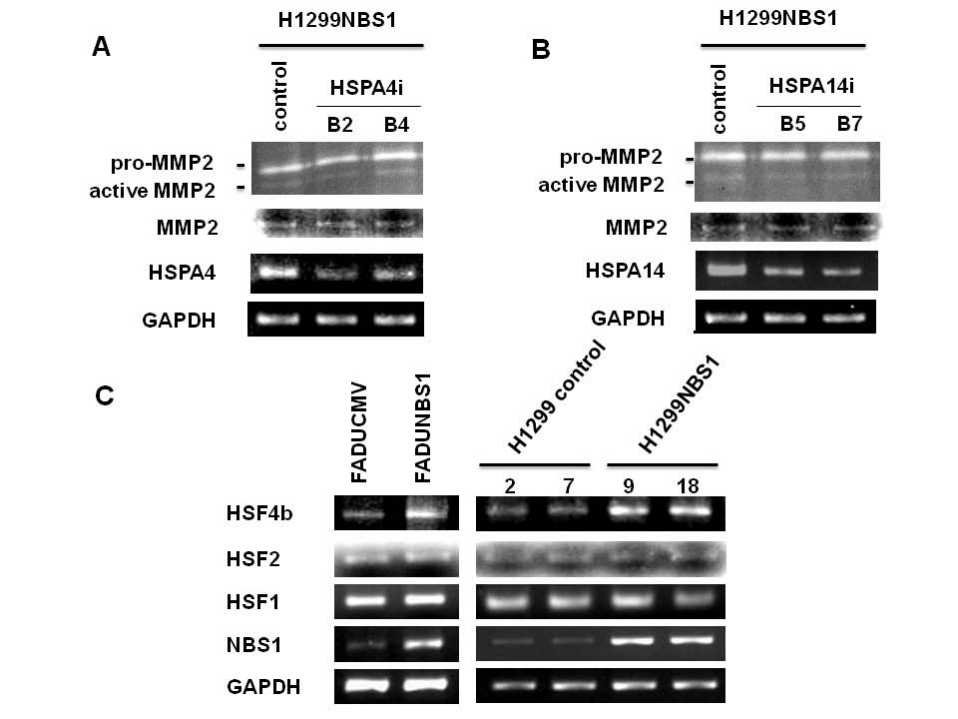
**MMP2 activity or expression was not affected by repression of HSPA4 or HSPA14 in H1299NBS1 cells and the correlation of HSF4b expression with HSPA4/HSPA14 expression**. **(A) & (B) **Gelatin zymography of the conditioned medium of H1299NBS1 control vs. H1299NBS1-HSPA4i clones **(A) **or H1299NBS1-HSPA14i clones **(B)**, which was shown in the first lane of each panel. Both pro-MMP2 and active MMP2 activity were stained. The second to the fourth lanes showed the RT-PCR analysis of MMP2, HSPA4/HSPA14, and GAPDH levels in H1299NBS1 control vs. H1299NBS1-HSPA4i clones **(A) **or H1299NBS1-HSPA14i clones **(B)**. **(C) **RT-PCR analysis showed the induction of HSF4b, but not HSF1 or HSF2, by NBS1 overexpression in two different cell lines (FADU control vs. FADUNBS1, H1299 control vs. H1299NBS1).

Since we previously showed that NBS1 overexpression induced epithelial-mesenchymal transition through the Snail/MMP2 pathway [17], we wanted to test whether MMP2 activity overlapped with the activity of HSPA4 or HSPA14. Gelatin zymography experiments showed that there was no decrease in pro-MMP2 or active MMP2 activity when either HSPA4 or HSPA14 were knocked down by siRNA (first lane of Figure 5A, B). There was no decrease in the mRNA levels of MMP2 in H1299NBS1 stable clones receiving siRNA against HSPA4 or HSPA14 (lower lanes of Figure 5A, B). This result suggests that the pathways of HSPA4 or HSPA14 did not overlap with the Snail/MMP2 pathway previously shown [17]. To screen for the upstream signaling which may induce the expression of HSPA4 or HSPA14, RT-PCR analysis of different heat shock transcription factors was performed. The result showed that the mRNA levels of HSF4b, but not HSF1 or HSF2, correlated with the expression of HSPA4 and HSPA14 (Figure 5C). This result suggests that HSF4b may be the transcription factor inducing the activation of HSPA4 or HSPA14 in NBS1 overexpressing cells.

## Discussion

Overexpression of NBS1 was previously shown to induce transformation through the PI 3-kinase/Akt pathway and epithelial-mesenchymal transition through the Snail/MMP2 pathway [11-13,17]. However, NBS1 may activate other pathways that promote EMT and metastasis since ~9% of metastatic head and neck cancer patient cases belonged to the NBS1(+)/Snail(-) group in our previous study [[17] and data not shown]. In this report, we demonstrated that NBS1 overexpression induced the expression of HSPA4 and HSPA14, two heat shock proteins. In addition, HSPA4 and HSPA14 contributed to the *in vitro *migration, invasion, and transformation activity using siRNA approaches, which is independent of MMP2 activity induced by NBS1 [17]. HSF4b could be the possible heat shock transcription factor which may cause the activation of HSPA4 and HSPA14. These results demonstrate the alternative pathway to induce *in vitro *metastatic and transformation activity when NBS1 is overexpressed. Although NBS1 overexpression was shown to induce PI 3-kinase/Akt activity [11-13], no putative Akt phosphorylation sites could be identified from the protein sequence of HSF4b. Since HSF4b was shown to be phosphorylated by ERK to increase its DNA binding activity [27], it will be interesting to test whether the ERK activity could be induced by NBS1 overexpression, leading to the phosphorylation of HSF4b.

HSPA4 and HSPA14 are two heat shock protein family members whose functions were not shown to be related to metastasis or transformation [18,20-25]. It is intriguing that siRNA mediated repression of either HSPA4 or HSPA14 caused a significant decrease in *in vitro *migration, invasion, and transformation activity. However, it appears that the role of HSPA4 and HSPA14 is overlapping since knockdown of both molecules did not further decrease the *in vitro *migration and invasion activity (additional file 2). Whether HSPA4 and HSPA14 regulate the same molecules to mediate metastasis remains to be explored. Our results uncovered the novel functions of these two heat shock proteins and delineated new roles of these two proteins in tumor metastasis and transformation. Increased HSPA4 levels were observed in hepatocellular carcinoma [19], which supports our observation.

## Conclusion

Our results indicate that NBS1 overexpression in cancer cells induces EMT and transformation through the activation of distinct pathways. This discovery provides valuable information for the diagnosis/prognosis and future target of anti-metastasis therapy in cancer patients.

## List of abbreviations

NBS: Nijmegen breakage syndrome; MRN: Mre11-Rad50-NBS1; HNSCC: head and neck squamous cell carcinoma; EMT: Epithelial-mesenchymal transition; HSP: heat shock protein; HSF: heat shock transcription factor; MMP: metalloproteinase; RT-PCR: reverse transcription-polymerase chain reaction

## Competing interests

The authors declare that they have no competing interests.

## Authors' contributions

CYW and CTL performed experiments, analyzed data and contributed equally to the work. MZW performed the experiments of additional file 2. KJW designed the experiments and wrote the manuscript. All authors read and approved the final manuscript.

## Supplementary Material

Additional file 1 **supplementary table 1**. the table contains sequences of oligonucleotides and primers used in the generation of pSUPER siRNA constructs and RT-PCR. Click here for file

Additional file 2 **supplementary figure 1**. Simultaneous knockdown of HSPA4 and HSPA14 did not further decrease the in vitro migration and invasion activity in H1299 cells. (A) RT-PCR analysis of H1299 cells with knockdown of HSPA4, HSPA14, or both. (B) The *in vitro *migration and invasion activity of H1299 cells with knockdown of HSPA4, HSPA14, or both. Click here for file
