# Supplementary material for: Induction of HSPA4 and HSPA14 by NBS1 overexpression contributes to NBS1-induced in vitro metastatic and transformation activity
Source: J Biomed Sci. 2011 Jan 6;18(1):1. doi: 10.1186/1423-0127-18-1 (PMC3022804; doi:10.1186/1423-0127-18-1)
Supplement: Additional file 1 — supplementary table 1. the table contains sequences of oligonucleotides and primers used in the generation of pSUPER siRNA constructs and RT-PCR. [file 1423-0127-18-1-S1.DOC]

**Supplementary Table 1. Sequences of oligonucleotides used to generate pSUPER-HSPA4i and pSUPER-HSPA14i and primers used in RT-PCR assays**

| Oligonucleotides used to generate pSUPER-HSPA4i **(target sequence)** | | | |
| --- | --- | --- | --- |
| HSPA4i-primerF | GATCCCC**GATCAGAATGCAAAGGAGG**TTCAAGAGA**CCTCCTTTGCATTCTGATC**TTTTTA | | |
| HSPA4i-primerR | AGCTTAAAAA**GATCAGAATGCAAAGGAGG**TCTCTTGAA**CCTCCTTTGCATTCTGATC**GGG | | |
| Oligonucleotides used to generate pSUPER-HSPA14i **(target sequence)** | | | |
| HSPA14i-primerF | GATCCCC**TTGGGAAGTGCCAACTGTT**TTCAAGAGA**AACAGTTGGCACTTCCCAA**TTTTTA | | |
| HSPA14i-primerR | AGCTTAAAAA**TTGGGAAGTGCCAACTGTT**TCTCTTGAA**AACAGTTGGCACTTCCCAA**GGG | | |
| RT-PCR primers | Position surrounding the transcription start site | sequence（5’🡪 3’） | Ampli-con length（bp） |
| HSF1-F | +1908 ~ +1935 | CCTCTGGTCAGGAGGGTCACCCTGGCCT | 214 |
| HSF1-R | +2093 ~ +2122 | TGCCTGTCTTGTCCGTCCATCCACTGTGTG |
| HSF2-F | +2258 ~ +2291 | GTGTACGTGAATGCTCGCTGTCTGATAGGGTTCC | 148 |
| HSF2-R | +2298 ~ +2235 | CAGTATGGTTCAAAAAAACACAAACCGGAAAACC |
| HSF4a-F | +1311 ~ +1340 | ctactgggcgaggtgcaggctttgcgggga | 374 |
| HSF4a-R | +1656 ~ +1684 | CCCTGGCCCTGTGAGGGCTAAGGCCCAAAT |
| HSF4b-F | +1311 ~ +1340 | ctactgggcgaggtgcaggctttgcgggga | 402 |
| HSF4b-R | +1684 ~ +1713 | CAGGGGATGGAGAGTCTTCTGGGATGTCAG |
| HSPA1A-F | +2132 ~ +2159 | CCAAGATTGCTGTTTTTGTTTTGGAGCT | 145 |
| HSPA1A-R | +2248 ~ +2276 | CTGCATGTAGAAACCGGAAAAAAAGCAAG |
| HSPA4-F | +1 ~ +25 | GCTCTGGTGCTGCGGCTCCGCTCTC | 167 |
| HSPA4-R | +144 ~ +167 | CTTGCGGTGTCCACGCGAGCGCCT |
| HSPA8-F | +373 ~ +397 | GGCAGGCCCAAGGTCCAAGTAGAAT | 398 |
| HSPA8-R | +746 ~ +770 | TCTCCACCCAAGTGGGTGTCTCCAG |
| HSPA14-F | +901 ~ +925 | AATGCGCGAGCCATGATGAAATTAA | 402 |
| HSPA14-R | +1278 ~ +1302 | TTCCACCAACAGGTTTTCTTTCCCA |
| NBS1-F | +817 ~ +839 | AGAAATTGAGTTCCGCAGTTGTC | 574 |
| NBS1-R | +1369 ~ +1390 | GGGATTCTCATCTTAGCCAAAG |
| GAPDH-F | +108 ~ +127 | GAAGGTGAAGGTCGGAGTCA | 402 |
| GAPDH-R | +490 ~ +509 | TTCACACCCATGACGAACAT |
