# Supplementary figures and images for: Induction of HSPA4 and HSPA14 by NBS1 overexpression contributes to NBS1-induced in vitro metastatic and transformation activity
Source: J Biomed Sci. 2011 Jan 6;18(1):1. doi: 10.1186/1423-0127-18-1 (PMC3022804; doi:10.1186/1423-0127-18-1)

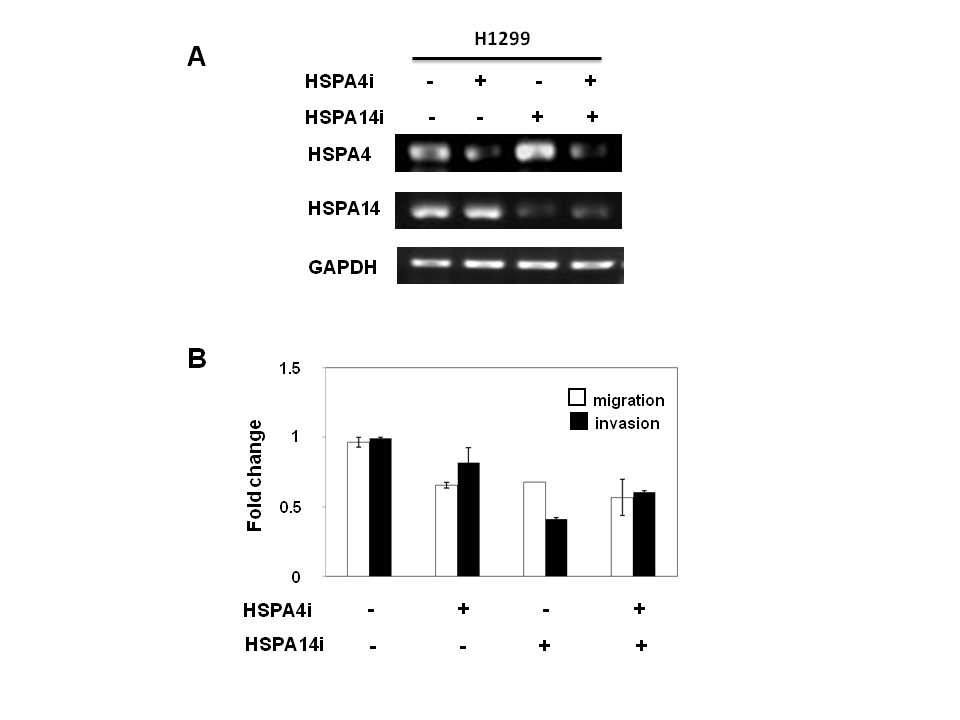

Supplement: Additional file 2 — supplementary figure 1. Simultaneous knockdown of HSPA4 and HSPA14 did not further decrease the in vitro migration and invasion activity in H1299 cells. (A) RT-PCR analysis of H1299 cells with knockdown of HSPA4, HSPA14, or both. (B) The in vitro migration and invasion activity of H1299 cells with knockdown of HSPA4, HSPA14, or both. [file 1423-0127-18-1-S2.TIFF]
